# Targeting adenosine for cancer immunotherapy

**DOI:** 10.1186/s40425-018-0360-8

**Published:** 2018-06-18

**Authors:** Robert D. Leone, Leisha A. Emens

**Affiliations:** 1 Bloomberg~Kimmel Institute for Cancer Immunotherapy, Sidney-Kimmel Comprehensive Cancer Center, Department of Oncology, Johns Hopkins University School of Medicine, Baltimore, MD 21287 USA; 2 Johns Hopkins University School of Medicine, 1650 Orleans Street, Room 409, Cancer Research Building 1, Baltimore, MD 21231 USA

**Keywords:** Adenosine, A2a receptor, Checkpoint blockade, PD-1, Immunotherapy, CD39, CD73, CTLA-4

## Abstract

Immune checkpoint antagonists (CTLA-4 and PD-1/PD-L1) and CAR T-cell therapies generate unparalleled durable responses in several cancers and have firmly established immunotherapy as a new pillar of cancer therapy. To extend the impact of immunotherapy to more patients and a broader range of cancers, targeting additional mechanisms of tumor immune evasion will be critical. Adenosine signaling has emerged as a key metabolic pathway that regulates tumor immunity. Adenosine is an immunosuppressive metabolite produced at high levels within the tumor microenvironment. Hypoxia, high cell turnover, and expression of CD39 and CD73 are important factors in adenosine production. Adenosine signaling through the A2a receptor expressed on immune cells potently dampens immune responses in inflamed tissues. In this article, we will describe the role of adenosine signaling in regulating tumor immunity, highlighting potential therapeutic targets in the pathway. We will also review preclinical data for each target and provide an update of current clinical activity within the field. Together, current data suggest that rational combination immunotherapy strategies that incorporate inhibitors of the hypoxia-CD39-CD73-A2aR pathway have great promise for further improving clinical outcomes in cancer patients.

## Background

The ability to regulate the strength and duration of an immune response is a critical aspect of immunity. To this end, an array of mechanisms has evolved that protect normal tissue from autoimmune attack or an over-exuberant response to pathogens. These regulatory mechanisms include selection of immune cell populations by thymic education as well as molecular pathways that regulate them. Interestingly, many inhibitory signals, or “immune checkpoint pathways,” are specifically upregulated on lymphocytes at the time of activation, suggesting that establishing regulatory signals to counter activation is an intrinsic aspect of an evolving immune response. While checkpoint pathways are critical to controlling physiologic inflammatory responses, they are also highly active in human cancers and provide a mechanism for tumors to evade the anti-tumor immune response. Recently, great strides have been made in treating human cancers through blockade of immune checkpoint pathways to unleash a latent immune response [1]. First demonstrated in the treatment of patients with advanced melanoma, blockade of the CTLA-4 and PD-1 immune checkpoint pathways in the clinic generates an unprecedented survival benefit in some cancer patients. The use of PD-1 and CTLA-4 blockade is rapidly expanding to a growing list of cancers. Significant gains have been achieved in non-small cell lung cancer, renal cell carcinoma, bladder cancer, head and neck cancer, Merkel cell cancer, and micro-satellite unstable disease [2, 3]. While these successes represent an undeniable paradigm shift in cancer treatment, most patients still fail to respond to immune checkpoint therapies. Thus, significant efforts are underway to identify new targets that activate, unleash, or enhance the immune response against cancer. One critical mechanism of cancer immune evasion is the generation of high levels of immunosuppressive adenosine within the tumor microenvironment. This pathway presents several promising therapeutic targets for immunotherapy. In this review, we will examine the rationale for therapeutic blockade of this pathway, provide an overview of preclinical data, briefly describe ongoing clinical work, and assess potential future applications targeting the adenosine pathway in cancer immunotherapy.

### Adenosine signaling protects against excessive immunologic responses

**Fig. 1:**
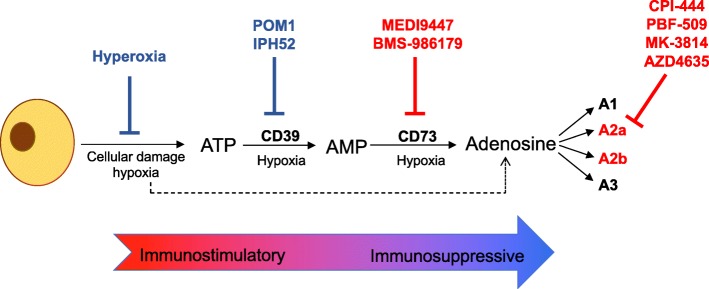
Extracellular Adenosine Creates a Highly Immunosuppressive Microenvironment. ATP in tumor tissue is released from cellular breakdown as well as vesicular and/or channel-mediated release. Adenosine is generated from enzymatic dephosphorylation of ATP, as well as direct release from tumor cells. While ATP functions as a danger signal for immune response, adenosine is an immunosuppressive metabolite, signaling largely through the A2aR and A2b receptors on a wide range of innate and adaptive immune cells. Inhibiting agents presently in clinical trials are depicted in red; agents targeting hypoxia and CD39 are in preclinical stage of development and are depicted in blue. Agents targeting the A2b receptor are in various stages of development, but are not depicted

In addition to thymic (ie, “central”) processes of positive and negative selection of effector and regulatory T cell (Treg) clones, peripheral control mechanisms are crucial in regulating immune responses. Inherent in the initial activation of an immune response is the upregulation of signaling pathways to provide negative feedback and protection from over-exuberant responses that might endanger normal tissues. Purinergic signaling is an important component of this peripheral immune regulation (Fig. 1) [4]. Adenosine and ATP are normally present at very low levels in extracellular fluids [5]. Inflammation, ischemia, or cancer can lead to the release of high levels of ATP through a variety of mechanisms, including transporter- or channel-mediated release, active vesicular exocytosis, and direct release through mechanical stress or cell destruction [6]. By signaling through purinergic receptors on a wide array of immune cells, extracellular ATP functions as a Danger-Associated Molecular Pattern (DAMP) to promote both innate and adaptive immune responses [4, 6]. During inflammation, however, extracellular ATP is progressively dephosphorylated by ectonucleotidases (most prominently CD39 and CD73), culminating in the formation of adenosine [7]. In contrast to the immune stimulatory properties of extracellular ATP, extracellular adenosine has a marked dampening effect on the immune response, suppressing effector cell function and stabilizing immunosuppressive regulatory cells [4, 7]. Notably, these ectonucleotidases CD39 and CD73 are highly expressed on cells within the TME, including stromal cells, tumor cells, infiltrating immune cells, and endothelial cells, and are further upregulated in response to hypoxia through HIF1α mediated mechanisms [7, 8]. Furthermore, both CD39 and CD73 are upregulated on Tregs in response to adenosine signaling itself [9–11]. This sets up a positive feedback loop, leading to exponential increases in adenosine production and dramatic suppressive activity within immune synapses formed between Tregs and dendritic cells DCs.

Extracellular adenosine activates cellular signaling pathways through one of four known G-protein-coupled adenosine receptors, A1, A2a, A2b, and A3 [12]. The A2a and A2b receptors are G-protein coupled stimulatory pathways that are upregulated in response to immune cell activation. Upon engagement of the A2a or A2b receptors, adenosine triggers increased adenylyl cyclase activity with concomitant increases in intracellular cAMP [13]. Through the activation of protein kinase A, cAMP has profound effects on a wide range of immune cells and processes [4]. The A2a receptor is a high-affinity receptor and is expressed on T cells and natural killer T (NKT) cells, monocytes, macrophages, DCs and natural killer (NK) cells, whereas the A2b receptor is a relatively low-affinity receptor most highly expressed by macrophages and DCs [4]. The A2aR is upregulated in macrophages in response to NF-κB, STAT1, and PPARγ and adenosine signaling, and A2aR activation suppresses secretion of neutrophil chemoattractants, thereby blunting the inflammatory response [4]. Adenosine signaling can also impede DC maturation, inhibiting their ability to direct effector cell differentiation. It also skews DCs to more suppressive phenotypes that secrete IL-10, TGFβ, arginase, and IDO [14]. In effector T cells, increased PKA activity secondary to A2aR signaling has a number of suppressive effects, including 1) inhibiting proximal TCR signaling by blocking LCK-dependent activation of ZAP70 [15, 16]; 2) suppressing multiple MAP kinases (ERK1, JNK) [15]; 3) inhibiting protein kinase C activity, which is critical for effector cell activation [4]; 4) activation of CREB mediated inhibition of NF-κB and nuclear factor of activated T cells (NF-AT) [17]. A2aR signaling in CD4 T cells reduces IL-2 secretion, which dampens expression of the costimulatory receptor CD28 [18]. In effector CD8 T cells, adenosine signaling through the A2aR inhibits cell proliferation, effector cytokine production (TNFα, INFγ, IL-2), and cytotoxicity [4, 19, 20]. A2a receptor signaling also stabilizes Tregs by increasing expression of FOXP3, their pathognomonic transcription factor [21]. Lastly, A2aR signaling on both effector and regulatory T cells triggers the increased expression of other immune checkpoint pathways, including PD-1, CTLA-4, and LAG-3 [4, 7, 19]. Thus, A2aR signaling may represent a master checkpoint pathway. Overall, by signaling through A2a and A2b receptors, adenosine generation in inflamed tissues couples the resolution of inflammation in response to tissue injury to a profound dampening of the immune response. This coupling of wound healing and immunosuppression, however, is maladaptive in malignancy and is a fundamental mechanism of cancer immune evasion. Accordingly, it is a high priority target for immunotherapeutic intervention.

### Adenosine pathway blockade in immunotherapy for cancer: preclinical data

Many of the factors that favor adenosine generation—tissue disruption, hypoxia, ectonucleotidase expression, and inflammation—are highly characteristic of the tumor microenvironment [5]. Significant work has thus been done in targeting various aspects of tumor-associated adenosine signaling as a means of enhancing the immune response to malignancy. These studies have generally focused on two primary aspects of immunosuppressive adenosine: 1) inhibition of adenosine production in the TME through targeting CD73 and/or CD39, and 2) the blockade of adenosine signaling through targeting the A2a and A2b receptors (Fig. 1). Given its high affinity and broad distribution, targeting the A2a receptor has garnered a particularly high level of interest as a novel immune checkpoint in cancer. The A2a receptor is also of great interest as many small molecule inhibitors have already been developed and tested in patients with a variety of neurological indications, particularly Parkinson’s Disease [22]. While these agents have generally been well tolerated (with no significant immune-related toxicities), clinical trials have not demonstrated significant or consistent efficacy of A2aR antagonists [22]. Although the failure to demonstrate efficacy of these agents has generally been ascribed to problems with complicated trial designs [23, 24], recent preclinical studies have demonstrated that high concentrations of A2aR antagonists can behave as agonists when interacting with complexes of the A2aR and dopaminergic D2 receptor [25]. More studies are needed to determine the best way to apply A2aR antagonists in neurologic diseases such as Parkinson’s Disease.

#### A2aR blockade in preclinical models

Seminal work by the Sitkovsky group and others over the past two decades has revealed the multitude of interactions between adenosine and distinct immune cells in the tumor microenvironment (TME) [26–28]. The Sitkovsky group was the first to show that several tumor lines, including a melanoma line and a T cell lymphoma line, were completely rejected in A2aR-null mice in a CD8-dependent manner [29]. They also demonstrated that pharmacologic blockade of the A2aR could enhance T cell mediated tumor regression in both a sarcoma model, and in the poorly immunogenic LL-LCMV tumor model [29]. Others demonstrated the ability of A2aR blockade to suppress metastatic potential in several syngeneic tumor models in mice, including the 4 T1 breast cancer model and the B16F10 melanoma model [30]. The Powell laboratory reported the ability of A2aR-null mice to reject EL4 T cell tumors, and additionally showed that treatment with anti-PD-1 enhanced tumor control in A2aR-null mice [31]. Subsequent studies also demonstrated that combining A2aR inhibition with blockade of the PD-1, TIM-3, or CTLA-4 pathways was effective in enhancing immune-mediated control in a variety of syngeneic tumor models [32–34]. Most recently, A2aR blockade in the setting of CAR T cells significantly improved effector function and anti-tumor responses in a murine model [35]. Notably, it has been reported that T cell-specific knockout of the A2aR in mouse models downregulated the IL-7 receptor in naïve CD8+ T cells, attenuating their lifespan [36, 37]. Despite this finding, an attenuated CD8+ T cell lifespan using pharmacologic A2aR blockade in mouse models has not been reported. However, the longevity of T cells in the setting of A2aR blockade should be monitored closely in clinical trials since the establishment of a robust memory response is a critical aspect of immunotherapy.

#### CD73 blockade in preclinical models

In addition to targeting the A2aR, preclinical studies have investigated the value of targeting the upstream regulators of extracellular adenosine in the tumor microenvironment. Stagg and others have reported a range of significant findings targeting CD73, with several studies reporting robust tumor rejection in CD73-null mice in a variety of syngeneic tumor models [38]. Other studies have uncovered an important contribution of CD73 expression to tumor metastasis, which is reduced in syngeneic tumor models of melanoma and prostate cancer in CD73-null mice [38, 39]. Interestingly, CD73 expression on both immune cells and non-immune cells play important roles [8]. These studies established that CD73 expression on infiltrating Tregs with concomitant adenosine production plays a crucial role in suppressing the effector function of antitumor CTLs and NK cells. Furthermore, CD73 expression on endothelial cells within the TME appears to attenuate T cell trafficking to tumors, and may also play a critical role in tumor cell migration during the metastatic process [8]. Subsequent studies blocking CD73 function with therapeutic antibodies recapitulated the phenotype observed with genetic deletion, namely, suppression of tumor growth and metastases [8, 40–42]. Tumor growth suppression was found to be immune-mediated and was lost in A2aR-null mice, implicating adenosine signaling through this receptor on immune cells as the critical pathway of tumor control [42]. Blockade with anti-CD73 antibodies in murine tumor models also confirmed the ability to suppress metastases and tumor cell migration, an effect that was independent of hematopoetic cell involvement [8, 41, 42]. These studies point to a non-catalytic role of CD73 expressed on tumor cells or endothelial cells that is critical to tumor cell adhesion, extravasation, and metastasis. Mechanistic studies in a mouse model of human breast cancer have demonstrated that anti-CD73 monoclonal antibody caused clustering and internalization of CD73 that interfered with its role in cellular adhesion and migration [41].

#### CD39 blockade in preclinical models

As discussed, CD39 ectonucleotidase activity provides the critical initial step in the degradation of extracellular ATP to immunosuppressive adenosine. While CD39 is highly expressed on tumor infiltrating immune cells—particularly Tregs, effector T cells, and myeloid cells—like CD73, it is also often expressed on tumor-associated endothelium as well as tumor cells themselves [20, 43–46]. Early preclinical studies by the Robson lab showed that CD39 null mice were resistant to tumor metastases in B16/F10 mouse melanoma model, as well as the MCA-38 colorectal model [47]. Interestingly, metastasis was partially dependent on CD39 expression on both immune cells (particularly Tregs) as well as nonhematopoeitic cells. While CD39 expression on Tregs was crucial in suppressing NK cell antitumor activity, CD39 expression on tumor and endothelial cells, was found to be important in promoting angiogenesis and metastatic tumor spread [48, 49]. The Robson group subsequently demonstrated that overexpression of CD39 in tumor bearing mice led to increased liver metastases of MC-26 mouse colorectal tumors [50]. Additionally, pharmacological blockade of CD39 activity with the novel NTPDase inhibitor sodium polyoxotungstate (POM1) improved antitumor immunity and decreased metastatic spread in several tumor models [49]. Underscoring its importance in both immune and non-immune based cancer growth, blockade of CD39 enhanced the immune cell effector response to human ovarian cancer cell lines and follicular lymphoma cells in vitro*,* and the survival of NOD mice in patient-derived sarcoma models [51–53]. Overall, through its critical role in the degradation of extracellular ATP to adenosine and its broad range of expression on endothelium, immune cells, and cancer cells, CD39 has demonstrated multifunctional potential as a target for immunotherapy in cancer. Outside of its expression and suppressive effects on immune cells, the role of CD39 in angiogenesis and endothelial function are likely also crucial aspects of its effect on tumor growth and metastatic spread.

### The clinical significance of the adenosine pathway in cancer patients

#### CD73 as a biomarker

CD73 expression within the tumor microenvironment has been studied as a prognostic biomarker for clinical outcomes in several tumor types, including breast cancer, lung cancer, ovarian cancer, kidney cancer, gastric cancer, prostate cancer, urothelial cancer, uterine cancer, melanoma, and head and neck cancers (Table 1) [2, 54–64, 66]. The vast majority of these studies have demonstrated a statistically meaningful correlation between high CD73 expression and unfavorable clinical outcomes. This is consistent with the role of adenosine as an immunosuppressive metabolite. However, there have also been reports of CD73 expression predicting a favorable disease course, especially in some early stage disease states, including studies of patients with urothelial carcinoma, endometrial carcinoma, and breast cancer [61–64]. To this end, it has been theorized that CD73-derived adenosine may act as a barrier in the vascular endothelium, mitigating the metastatic process. This biology will be particularly important to consider as clinical trials testing CD73 antagonists begin to include patients with earlier stage (ie, not widely metastatic) disease.

**Table 1:** CD73 and CD39: Predictive and Prognostic Biomarkers

	Tumor Type	Findings	# of Patients	Study Author
CD73				
Negatively prognostic	NSCLC (stage I-III)	High CD73 expression was an independent risk factor for decreased overall survival and dereased recurrence-free survival	642	Inoue, et al. [54]
	Prostate Cancer	CD73 expression in normal tissue was a negative prognostic factor for prostate-infiltrating CD8(+) cells. However, high expression of CD73 in tumor stroma was associated with longer recurrence-free survival	285	Leclerc, et al. [55]
	Breast Cancer (Triple Negative)	CD73 expression is associated with anthracycline resistance and poor prognosis	6000	Loi, et al. [56]
	High-Grade Serous Ovarian Cancer	High levels of CD73 are associated with shorter disease-free survival and overall survival	1581	Gaudreau, et al. [57]
	Colorectal Cancer (stage I-IV)	High expression of CD73 predicts poor survival	223	Wu, et al. [58]
	Gastric Cancer (stage I-IV)	High expression CD73 is associated with lowered overall survival	68	Lu, et al. [65]
	Melanoma (Stage IV)	High soluble CD73 activity was associated with poor overall survival and poor progression-free survival	37	Morello, et al. [59]
	Head and Neck Cancer (stage I-IV)	High levels of CD73 are associated with reduced overall survival	162	Ren, et al. [60]
	Renal Cell Cancer (Stage I-IV)	High expression of CD73 is associated with disease progression and shortened overall survival	189	Yu, et al. [2]
Positively prognostic	Nonmuscle-Invasive Urothelial Bladder Cancer	High CD73 iactivity was associated with favorable clinicopathological features. Furthermore, predicts better outcome in the subgroup of pTa and pT1 tumors.	174	Wettstein, et al. [61]
	Breast Cancer (stage I-III)	CD73 expression strongly correlated with longer disease-free survival and overall survival	136	Supernat, et al. [62]
	Endometrial Carcinoma (endometrial endometrioid carcinomas, Grade 1–3) and nonendometrioid uterine papillary serous carcinomas	CD73 is markedly downregulated in poorly differentiated and advanced-stage disease compared with levels in normal endometrium and low-grade tumors	49	Bowser, et al. [64]
	Colorectal cancer (Stage IV)	High CD73 expression was associated with longer progression free survival from cetuximab treatment in patients with KRAS-WT and KRAS-mutant tumors	238	Cushman, et al. [63]
CD39				
Negatively prognostic	Gastric Cancer (stage I-IV)	High CD39 expression is a predictor of poor outcome following radical resection	101	Cai, et al. [69]
	Hepatocellular carcinoma	High CD39 expression is an independent indicator of decreased overall suvival after radical resection	324	Cai, et al. [70]
	Chronic lymphocytic leukemia	CD39 expression on CD4+ lymphocytes are increased in the peripheral blood of patients with CLL and correlates with advanced stage of disease	62	Perry, et al. [71]

#### CD39 as a biomarker

Like CD73, CD39 is expressed on both infiltrating immune cells, as well as on cancer cells themselves in a range of human cancers, including lung cancer, melanoma, pancreatic cancer and lymphoma [66–68], and studies have correlated high CD39 expression with unfavorable outcomes (Table 1) [69–71]. Clinical trials are underway to further define the importance of CD39 expression in the tumor microenvironment in ovarian and breast cancer in response to chemoradiation and anesthesia, respectively (Table 2). These data, along with more detailed correlative studies defining the prognostic and predictive value of CD39 expression on the full range of distinct cell types within the tumor microenvironment will provide important insights into CD39 as a clinically relevant therapeutic target.

**Table 2:** Overview of clinical trials investigating targets within the adenosine signaling pathway in cancer

Molecular Target	ClinicalTrials.gov Identifier	Pharmaceutical Supplier	Agent	Design Overview			
Interventional Trials							
A2aR	NCT02655822	Corvus	CPI-444	Phase 1/1b	single agent and in combination with Atezolizumab	allows prior PD-1/PD-L1	advanced solid malignancies
	NCT02403193	Pablobio (Novartis)	PBF-509	Phase 1/1b	single agent and in combination PDR001 (anti-PD1)	allows prior PD-1/PD-L1	non-small cell lung cancer
	NCT03099161	Merck	MK-3814	Phase 1	single agent and in combination with Pembrolizumab(anti-PD-1);	allows prior PD-1/PD-L1	advanced solid malignancies
	NCT02740985	AstraZeneca	AZD4635	Phase 1	single agent and in combination with Durvalumab (anti-PDL-1)	allows prior PD-1/PD-L1	advanced solid malignancies
CD73	NCT02503774	Medimmune	MEDI9447	Phase 1	combination with Durvalumab (anti-PDL-1) (no single agent)	allows prior PD-1/PD-L1	advanced solid malignancies
	NCT03267589	Medimmune	MEDI9447	Phase 2	single agent and in combination with Durvalumab (anti-PDL-1), Tremelilumab (anti-CTLA4), MEDI 0562 (anti-OX40)	allows prior PD-1/PD-L1	relapsed ovarian cancer
	NCT02754141	Bristol-Meyers-Squibb	BMS-986179	Phase 1/2a	single agent and in combination with Nivolumab (anti-PD-1)	allows prior PD-1/PD-L1	advanced solid malignancies
Noninterventional Trials^a^							
CD39/CD73	NCT03255252	Assessment Study to Evaluate Specific Immune Response in Locally Advanced Cervix Cancer After Radio-chemotherapy					
	NCT02567929	Assessment of the Anesthetic Effect on the Activity of Immune Cell in Patients With Breast Cancer					

^a^“Noninterventional trials” refers to interventions targeting the adenosine-CD39-CD73-A2aR pathway, these trials may be otherwise interventional in terms of other treatment modalities, such as chemotherapy or radiation

### Clinical trials of adenosine pathway blockade in cancer

Given the well-established clinical benefit of immune checkpoint blockade in cancer therapy and the promising preclinical activity of adenosine pathway blockade discussed, there is significant interest in translating adenosine-A2aR pathway blockade to the clinical setting. Several agents that block distinct targets along the adenosinergic pathway are presently in early phase clinical trials that evaluate their safety and clinical activity as both single agents and in combination.

#### Clinical application of A2aR blockade

Several small molecule inhibitors of the A2aR have previously been evaluated in phase 3 clinical trials for neurologic indications. While these agents demonstrated limited efficacy for Parkinson’s Disease or adult attention deficit hyperactivity disorder (ADHD), their safety profile and tolerability was excellent. Significant immune-related toxicities similar to those observed with anti-CTLA-4 and anti-PD-1 therapies have not been reported with single agent A2aR blockade. There are currently four agents targeting the A2a receptor for cancer immunotherapy in Phase 1 trials, including CPI-444 (Corvus), PBF-509 (Novartis/Pablobiofarma), MK-3814 (Merck), AZD4635 (AstraZeneca/Heptares) (Table 2). Each of these trials includes cohorts receiving monotherapy as well as A2aR blockade in combination with blockade of the PD-1/PD-L1 pathway. Data have so far only been reported for CPI-444 (Corvus).

A phase 1a/1b study is evaluating the safety and clinical activity of CPI-444 (Corvus) alone and in combination with Atezolizumab (Genentech) in patients with advanced solid tumors. It uses a 2-step design, where the first step evaluates dose and schedule in a variety of advanced cancers, and the second step further tests safety and clinical activity at the optimal dose and schedule in expansion cohorts of advanced melanoma, non-small cell lung cancer (NSCLC), triple negative breast cancer (TNBC), and renal cell carcinoma (RCC). Step 1 enrolled and treated 48 subjects [72]. The median number of prior systemic therapies was 4, and over 50% of patients had received prior PD-1/PD-L1 therapy. The median exposure to CPI-444 was 8 weeks. The most common adverse events were nausea and fatigue, and only one dose-limiting toxicity was noted (reversible grade 3 autoimmune hemolytic anemia). The optimal dose of CPI-444 was defined as 100 mg bid continuous. The disease control rate (DCR), defined as patients with a complete response (CR), partial response (PR) or stable disease (SD), was 45% in the overall patient population. Clinical activity was observed in all tumor types both as a single agent and in combination with atezolizumab, and appeared somewhat higher in RCC and NSCLC [73]. The majority of patients had SD, and 2 additional patients had a PR—both of these patients were on single agent CPI-444. Early biomarker analyses were reported on tissues donated by 80 patients [74]. Higher levels of A2AR, CD73, and CD39 were noted in patients who had received prior PD-1/PD-L1 therapy than those who had not, suggesting adenosine signaling may facilitate immune escape in these patients. Furthermore, expression of both CD73 and A2AR was identified as a potential predictive biomarker of response, and clinical activity was associated with expression of adenosine pathway genes in the tumor. CPI-444 induced CD8+ T cells infiltration into tumors, interferon-γ-dependent gene expression, and a T helper type 1 signature. TCR analyses showed that shared clonotypes expanded in matched post-dose PBMCs and tumor consistent with trafficking of T cells in response to CPI-444 treatment. This trial continues to accrue.

#### Clinical investigations of CD73 and CD39

Two monoclonal antibodies that inhibit CD73 are currently under investigation in early phase clinical trials (Table 2). A Phase 1/2a trial designed to assess the safety and efficacy of CD73 blockade with MEDI9447 (Medimmune) in combination with anti-PD-L1 therapy in patients with advanced solid tumors is presently recruiting, and interim results have not yet been disclosed. A second study is testing a variety of combinations of MEDI9447 (anti-CD73) with other monoclonal antibody immune checkpoint modulators: Durvalumab (anti-PD-L1), Tremelilumab (anti-CTLA-4), and MEDI0562 (anti-OX40). This Phase 2 study is designed to assess efficacy in the management of relapsed ovarian cancer. Bristol Meyers Squibb (BMS) is also conducting a clinical trial testing a CD73 antagonist (BMS986179) alone and with nivolumab in various solid tumors. The development of inhibitors of CD39 for cancer therapy is underway, but none have yet entered the clinic.

### Looking forward

With additional preclinical and clinical study, the application of inhibitors of the C39-CD73-A2aR pathway will be broadened and refined. The efficacy of combination regimens with other immune checkpoint inhibitors has been established in preclinical studies and evaluation in clinical studies has begun. Furthermore, upstream/downstream targeting of both the A2aR and CD73 has been shown to be efficacious in preclinical models, with each target likely engaging distinct immune mechanisms. Combination studies of A2aR and anti-CD73 antibodies are beginning to accrue. Recent preclinical studies showing the benefit of combining CAR T cell therapy with A2aR blockade have also been instructive [35], and clinical trials studying such regimens are on the horizon. Given that adenosine generation is dependent on both hypoxic conditions and cell turnover, blockade of this pathway should have value in combination with therapies that promote hypoxia and cell death within the TME. These modalities include radiation therapy, which generates hypoxic conditions, as well as chemotherapeutic agents, especially those that increase ATP release (i.e. “immunogenic chemotherapy”). Interestingly, Sitkovsky et al. demonstrated that hyperoxic conditioning of tumor-bearing mice slowed tumor growth [75]. This environmental manipulation effectively de-fuses the A2aR pathway at its most upstream modulator, hypoxia. It remains to be seen whether such a regimen can be adapted in a clinical setting [76]. Though beyond the scope of this review, there is also substantial interest in targeting the adenosine-A2b receptor for immunotherapy. The A2b receptor has a profound influence on innate immune cell function, and inhibitors specific for it are now entering Phase I clinical studies. The diversity of immune mechanisms mediated by adenosine signaling likely foreshadows a broad range of applications in the clinical arena.
